# Bivalent mRNA vaccine booster enhances immunity against XBB.1.5 more effectively than breakthrough infection in K18-hACE2 mice

**DOI:** 10.1016/j.isci.2025.113479

**Published:** 2025-09-01

**Authors:** Marissa E. Linger, Sytze H.T. Jorritsma, Jonna Bloeme-ter Horst, Jessika C. Zevenhoven-Dobbe, Finn Rijlaarsdam, Emil Colstrup, Macha Beijnes, Jutte J.C. de Vries, Ramon Arens, Rajagopal Murugan, Sebenzile K. Myeni, in collaboration with BREAK COVID group

**Affiliations:** 1 Leiden University Center of Infectious Diseases (LUCID), Leiden University Medical Center, Leiden, the Netherlands; 2 Department of Immunology, Leiden University Medical Center, Leiden, the Netherlands

**Keywords:** Immunology, Virology

## Abstract

The rapid emergence of SARS-CoV-2 Omicron subvariants, including the highly immune-evasive XBB.1.5 lineage, continues to challenge vaccine effectiveness. We evaluated immune responses and protection against XBB.1.5 challenge in K18-hACE2 mice receiving two or three doses of a bivalent mRNA vaccine (encoding Wuhan-1 and Omicron BA.4/5 spike) or two doses followed by intranasal XBB.1.5 exposure. Homologous boosting with bivalent vaccine induced stronger neutralizing antibody and cellular immune responses than breakthrough infection against the antigenically distant XBB.1.5 variant. A two-dose series alone protected mice from severe disease, but boosting further enhanced protection, reducing lung viral RNA and inflammation. Although productive infection following intranasal exposure was not confirmed, enhanced responses and viral control suggest reactivation of immune memory. Overall, homologous boosting with the bivalent vaccine enhances cross-variant immunity and protects against XBB.1.5 in a naive mouse model, supporting the utility of updated vaccines in improving immune breadth and protection against emerging SARS-CoV-2 variants.

## Highlights

• Bivalent mRNA vaccination protects mice against immune-evasive SARS-CoV-2 XBB.1.5
• Bivalent boosting elicits stronger immune responses than XBB.1.5 intranasal exposure
• Booster vaccination reduces lung viral RNA and inflammation after XBB.1.5 challenge

## Graphical abstract

**Figure:**
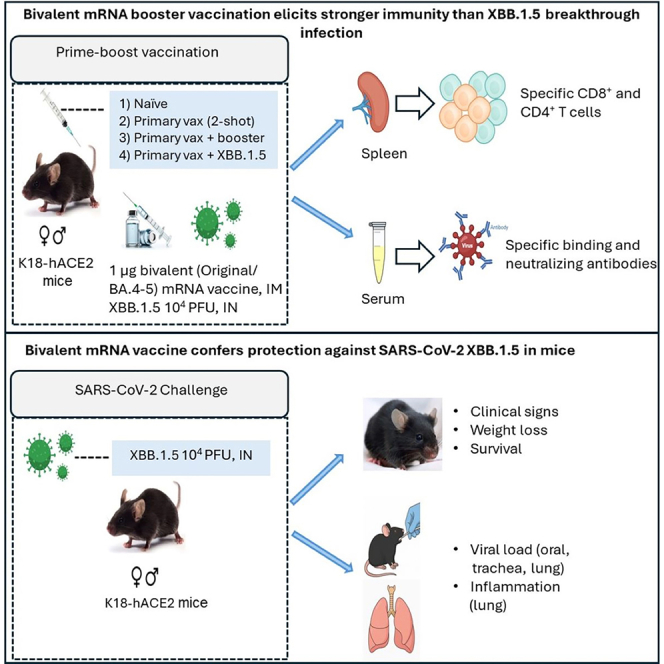

## Introduction

Since its emergence in December 2019, severe acute respiratory syndrome coronavirus 2 (SARS-CoV-2) has rapidly evolved into a diverse array of lineages.^1^^,^^2^ The recombinant Omicron XBB sublineages, which emerged in 2023, highlighted the virus’s ability to evolve enhanced transmissibility and evade immunity conferred by prior infections or vaccinations.^3^^,^^4^ This evolutionary shift facilitated the development of monovalent booster vaccines, including the XBB.1.5 monovalent mRNA vaccine, which was introduced in mid-2023, following the initial deployment of bivalent boosters in September 2022.^5^^,^^6^^,^^7^^,^^8^ Bivalent boosters incorporating mRNAs for the wild-type Wuhan-Hu-1, D614G, and Omicron spike proteins have been recommended as a single booster following the primary vaccination series and the monovalent booster with the original vaccine. These boosters have demonstrated the ability to elicit stronger neutralizing responses against many Omicron variants, though their effectiveness against XBB.1 strains remains limited.^9^^,^^10^^,^^11^ Several studies have shown that bivalent boosters generate more robust and broader neutralizing antibody responses than ancestral monovalent boosters; however, direct comparisons between bivalent vaccines and more recent variant-specific monovalent boosters (e.g., XBB.1.5 or KP.2) remain limited, and the relative efficacy of such boosters compared to prior infections is still being evaluated.^5^^,^^12^^,^^13^^,^^14^^,^^15^

Despite the ability of mRNA-based vaccines to be rapidly updated to target prevalent circulating lineages, new divergent strains often emerge before these updated vaccines become widely available, as evidenced by the introduction of the XBB.1.5 vaccine and the subsequent emergence of the JN1 and KP.2 lineages of Omicron sublineages.^14^^,^^16^ The ongoing surge in breakthrough infections, the widespread circulation of numerous of SARS-CoV-2 variants, and the persistent threat of future outbreaks driven by viral evolution underscore the urgent need for alternative vaccination strategies and booster vaccines capable of inducing a broad-spectrum immune response. However, widespread prior infections and vaccinations complicate the assessment of bivalent vaccine effectiveness against circulating variants, particularly across different vaccination regimens and in comparison to immunity from prior infections.^7^ As a result, preclinical animal studies remain essential for assessing the immunogenicity and protective efficacy of updated vaccines in naive models.

In this study, we evaluated the protective activity of the Comirnaty bivalent (Original/Omicron BA.4-5) mRNA vaccine against the SARS-CoV-2 XBB.1.5 strain in K18-hACE2 transgenic mice, both before and after boosting with the same vaccine or intranasal XBB.1.5 infection. We sought to evaluate the immune responses elicited by the bivalent formulation when used as both a primary series and a booster. This strategy allowed us to isolate the contributions of a vaccine containing both ancestral and Omicron spike components in a controlled immunological context, naive system, free from prior imprinting and minimizing confounding effects from prior ancestral strain exposure.^17^^,^^18^ Our study thus focused on the intrinsic boosting potential of the bivalent vaccine against the highly immune-evasive XBB.1.5 variant, as compared to infection-induced boosting with the same strain. This model provides insight into bivalent vaccine efficacy in populations with limited or no prior exposure, such as children born during the Omicron era. We measured the resulting humoral and cellular immune responses and assessed the antiviral protective effects of the bivalent vaccine when compared to a natural infection with the XBB.1.5 strain. In contrast with early Omicron subvariants that showed attenuated phenotype in mice,^19^^,^^20^^,^^21^ our data show that XBB.1.5 is pathogenic and leads to a sublethal infection in K18-hACE2 mice. We show that boosting with bivalent vaccine after the primary series induced stronger neutralizing antibody and cellular immune responses than XBB.1.5 infection, against both preceding Omicron and, strikingly, the XBB.1.5 strain. Our data further demonstrate that while a primary vaccination is sufficient in protecting mice from disease, boosting with the bivalent vaccine is required for enhanced immunogenicity, limiting breakthrough infections and reduced lung inflammation when challenged with the SARS-CoV-2 XBB.1.5 strain.

## Results

### Boosting with the bivalent mRNA vaccine enhances neutralizing antibody responses against SARS-CoV-2 XBB.1.5 in K18-hACE2 mice

**Figure 1:**
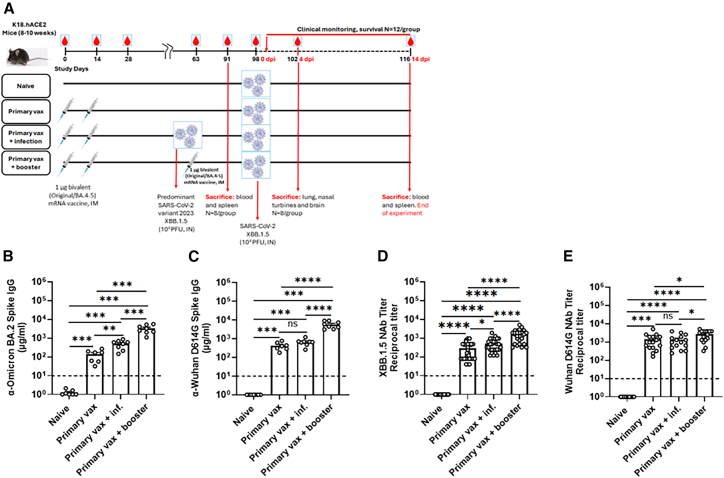
Serum antibody responses in K18-hACE2 mice following immunization with the bivalent (original and Omicron BA.4/BA.5) mRNA COVID-19 vaccine (A–E) Study design: scheme of immunization, blood collection, and virus challenge. Groups of male and female K18-hACE2 mice (8–10 weeks old) were immunized via an intramuscular route with the bivalent mRNA COVID-19 vaccine, sera were collected at 91 days after immunization (before challenge), and serum spike-specific IgG (B and C) levels were determined (*n* = 8 per group). Serum neutralizing antibody titers were determined against the indicated SARS-CoV-2 isolate across a broader set of animals, including those from the immunogenicity subset (used in B and C), as well as animals from challenge, fixed time point (day 4 post-infection), and survival groups (*n* = 28 per group) at 91 days after immunization (D and E). Bars show geometric mean values with geometric standard deviation and dotted lines indicate lower limit of detection (LOD). ∗*p* < 0.05, ∗∗*p* < 0.01, ∗∗∗*p* < 0.001, and ∗∗∗∗*p* < 0.0001; ns, not significant.

Since booster doses with the bivalent mRNA vaccine have been used in humans to broaden immunity against variants of SARS-CoV-2, including Omicron,^22^^,^^23^ we evaluated the immunogenicity and protective efficacy of the bivalent vaccine against SARS-CoV-2 XBB.1.5 variant in K18-hACE2 mice. Groups (*n* = 28 per group) of K18-hACE2 mice (8–10 weeks old) were immunized twice at a 2-week interval with 1 μg of the bivalent mRNA vaccine (two-dose series) or control (PBS—naive group; Figure 1A). Five weeks after the second dose, mice were boosted with either PBS (primary vax group), or received 1 μg of the bivalent mRNA vaccine (booster vax group, three-dose series) or intranasally with 10^4^ plaque-forming unit (PFU) SARS-CoV-2 XBB.1.5, the same dose used for final challenge (Figure 1A). Serum samples were collected at days 0, 14, 28, 63 (pre-boost), 91, and 98 (post-boost/pre-challenge) and day 102 and 116 (post-challenge). Four weeks (day 91) after booster vaccinations or XBB.1.5 infection, serum samples were analyzed for binding to the Omicron BA.2 (closely related to and used as a surrogate for XBB.1.5 at the time; Figure 1B) and Wuhan S protein (Figure 1C). Compared to the naive group, all vaccinated groups had higher serum IgG levels against both Wuhan and BA.2 Omicron S proteins (Figures 1B and 1C). Among the primary vaccinated mice, booster vaccination induced higher levels of anti-BA.2 S IgG antibodies than intranasal infection with SARS-CoV-2 XBB.1.5 virus (Figures 1B and 1C). Sera collected pre-challenge were also tested for neutralizing activity against Wuhan and XBB.1.5 strains. Mice boosted with the bivalent mRNA vaccine exhibited higher neutralizing responses against both Wuhan and XBB.1.5, compared to the primary vaccination group or the group infected with XBB.1.5 via intranasal infection (Figures 1D and 1E). As expected, in this mouse model and at the vaccine doses tested, boosting with the bivalent vaccine also induced stronger neutralizing antibody responses against the ancestral Wuhan strain compared to the XBB.1.5 variant (Figures 1D and 1E). Independent of vaccination and infection history, virus neutralizing titers broadly correlated with serum antibody titers across all groups. These data suggest that boosting with the bivalent mRNA vaccine after the primary vaccination series generates higher neutralizing antibody levels compared to prior infection with SARS-CoV-2 XBB.1.5.

### Boosting with the bivalent mRNA vaccine induces robust T cell responses against SARS-CoV-2

**Figure 2:**
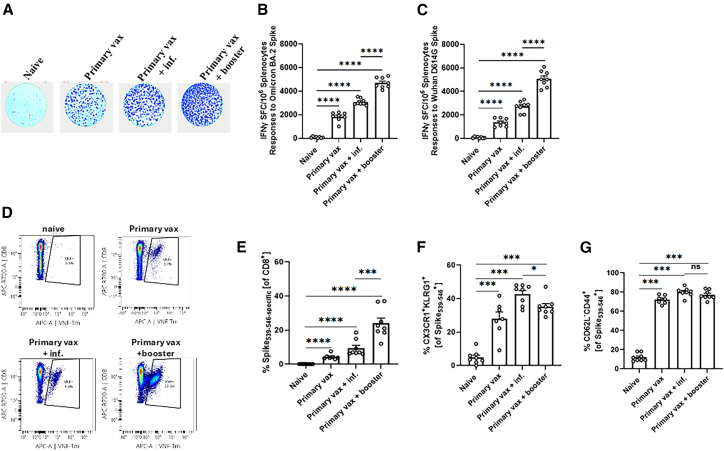
SARS-CoV-2 specific T cell immune responses in immunized mice K18-hACE2 mice were immunized and boosted as shown in Figure 1A. Splenocytes were collected 91 days after immunization (before challenge) for analysis of T cells by ELISpot and flow cytometry. (A) Qualitative representation of ELISpot results. (B and C) Magnitude of SARS-CoV-2-specific cell responses directed against Spike. IFN-γ was evaluated by an ELISpot assay from splenocytes derived from immunized mouse groups at 4 weeks post booster vaccination (before challenge). (D and E) Quantification of spike-specific tetramer^+^ CD8^+^ T cells in spleen tissues. Illustrating representative flow cytometry scatterplots and showing frequencies (D) and total cell numbers (E). (F and G) Effector-memory-like phenotype of VNF^+^CD8^+^ T cells based on their marker expression (CX3CR1^+^KLRG1^+^CD62L^−^CD44^+^). Bars show mean values and ±SEM. ∗*p* < 0.05, ∗∗*p* < 0.01, ∗∗∗*p* < 0.001, and ∗∗∗∗*p* < 0.0001; ns, not significant.

As effective vaccine immunity against SARS-CoV-2 variants has been reported to correlate with both antibody and T cell responses,^24^ we measured the levels of SARS-CoV-2-specific T cells in the vaccinated mice. Spike-specific T cell responses (using a spike peptide pool from Wuhan-Hu-1 and Omicron BA.2) in splenocytes harvested from K18-hACE2 mice (*n* = 8 per group), immunized with the primary series of the bivalent vaccine, and boosted with either PBS, the bivalent vaccine, or intranasally exposed to XBB.1.5, were quantified by ELISPOT (Figures 2A–2C) and flow cytometry (Figures 2D–2G). At pre-challenge, following the booster vaccinations or intranasal exposure with XBB.1.5, all primary and booster vaccinated/infected mice showed high numbers of interferon (IFN)-γ-secreting splenocytes compared to the naive, unvaccinated control group (Figures 2A–2C). Stronger T cell responses were observed after bivalent boosting against Omicron BA.2 or Wuhan spike peptides compared to a prior infection with SARS-CoV-2 XBB.1.5 or primary vaccination (without boosting; Figures 2A–2C). Furthermore, we used S-protein derived octameric peptide VNFNFNGL tetramers to identify and quantify conserved S-specific CD8 T cells.^25^^,^^26^^,^^27^ While all vaccinated groups induced an S-specific CD8^+^ T cell response (Figures 2D and 2E), boosting with the bivalent mRNA vaccine induced higher frequency of CD8^+^ T cell responses compared to an infection with SARS-CoV-2 XBB.1.5 or primary vaccination (Figures 2D and 2E). Independent of the vaccination/infection regimen, we observed that the S-specific CD8 T cells adapted effector-memory-like phenotype (CX3CR1^+^KLRG^+^), expressing tissue-residential markers (CD62L^−^CD44^+^; Figures 2F and 2G). In line with our observation of robust neutralizing antibody responses, boosting with the bivalent mRNA vaccine after primary vaccination induced stronger T cell responses compared to a prior intranasal infection with SARS-CoV-2 XBB.1.5.

### Bivalent mRNA vaccine protects mice from SARS-CoV-2 XBB.1.5 challenge

**Figure 3:**
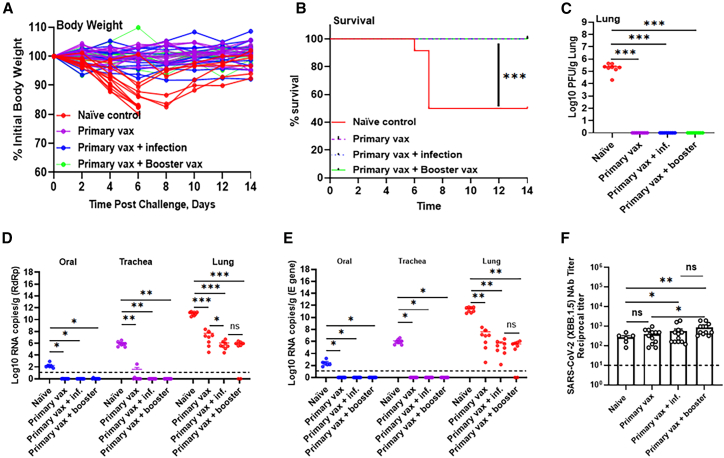
Protection against SARS-CoV-2 XBB.1.5 challenge after boosting with bivalent mRNA vaccine and natural infection in K18-hACE2 mice Male and female K18-hACE2 mice were immunized and boosted with the bivalent mRNA vaccine; and then, 5 weeks later, challenged via intranasal route with 10^4^ PFU of SARS-CoV-2 XBB.1.5. (A–F) Mice were monitored for 14 days after virus challenge for weight loss (A), and survival (B). Infectious virus units in the lung (C) and viral RNA levels in the lung, trachea, and oral swabs (D and E) at 4 days after SARS-CoV-2 XBB.1.5 challenge. Sera collected at 14 days post challenge and neutralizing antibody titers (F) were determined. Horizontal lines (C–E) and bars (F) show geometric mean values with geometric standard deviation (F), and dotted lines indicate lower limit of detection (LOD). ∗*p* < 0.05, ∗∗*p* < 0.01, ∗∗∗*p* < 0.001, and ∗∗∗∗*p* < 0.0001; ns, not significant.

Given the strong immunogenicity of the bivalent vaccine in our study, we next tested the *in vivo* protective efficacy of booster vaccination with either the bivalent mRNA vaccine or prior infection with XBB.1.5 in K18.hACE2 mice. Five weeks post-boost or exposure to XBB.1.5 infection, mice were challenged with a dose of 10^4^ PFU of XBB.1.5 and monitored for weight loss, survival, and viral replication at 4 days post-infection (dpi), the peak of viral burden in this model. While the challenge of naive (unvaccinated control) mice with XBB.1.5 resulted in rapid and significant weight loss by day 6 post challenge, no weight loss or ill-health were observed in any of the vaccinated groups, regardless of whether they were boosted with the bivalent vaccine or prior intranasal infection with XBB.1.5 (Figure 3A). Moreover, all animals vaccinated with the bivalent vaccine survived until the end of the experiment (14 dpi) while 50% of the naive control mice succumbed to infection before day 7 (Figure 3B). Notably, while over 90% (11/12) of the naive animals rapidly lost weight and exhibited disease symptoms, some of these animals began to recover and regain weight between days 6–8 (Figure 3D), which accounts for the 50% survival rate (Figure 3B). Compared to earlier Omicron subvariants, including BA.1, BA.2, and the more recent BA.4 and BA.5 strains, which are less pathogenic in mice,^28^^,^^29^^,^^30^ we show that XBB.1.5 is pathogenic in mice, resulting in a sublethal infection (Figures 3A and 3B). To measure the degree of vaccine-mediated protection, we measured infectious virus in the lungs (Figure 3C) and viral RNA levels in the oral cavity, trachea, and lung at 4 dpi (Figures 3D and 3E). While high levels of infectious virus were observed in unvaccinated naive animals, no detectable infectious virus (lower limit of detection, 10 PFU/mL) was found in any of the vaccinated animals, regardless of their booster status or infection history (Figure 3C). In the upper respiratory tract (oral and trachea cavities), all vaccinated mice showed reduced levels of both genomic and subgenomic viral RNA compared to naive controls (Figures 3D and 3E). Boosting with the bivalent vaccine or a prior intranasal infection with XBB.1.5 resulted in similar viral RNA levels in the lungs, that were ∼6 log copies/g lower than in the naive control lungs (Figures 3D and 3E). In contrast, mice that received only a primary vaccination, conferred a significantly lower viral RNA reduction in lungs, ∼4 log copies/g lower when compared to the naive animals, versus the ∼6 log reduction in boosted animals (Figures 3D and 3E). We also measured neutralizing antibody activity in sera against XBB.1.5 fourteen days post-challenge (Figure 3F). Among the naive control group, approximately 50% of the animals succumbed to XBB.1.5 challenge. Of the survivors, only a subset developed detectable neutralizing antibodies, and these were generally of low magnitude (Figure 3F). In contrast, all vaccinated groups exhibited robust neutralizing responses. Animals that received primary vaccination or were infected with XBB.1.5 after the primary vaccination series showed modest neutralizing responses, in contrast to the greater activity measured in animals that received a bivalent booster (Figure 3F). Collectively, these data indicate that immunization with the bivalent mRNA vaccine protects mice from a severe disease caused by XBB.1.5 infection and suggest that boosting with the bivalent vaccine or intranasal XBB.1.5 exposure after primary vaccination significantly enhances protection against XBB.1.5 strain, as evidenced by lower lung viral RNA levels compared to animals that received only two doses of vaccine (*p* < 0.01). To quantify the relationship between immune responses and protection, we performed Spearman correlation analyses (Tables S1 and S2), which revealed significant inverse correlations between lung viral RNA levels and both neutralizing antibody titers and T cell responses. These data demonstrates that antibody (Figure 1) and T cell responses (Figure 2) predicted the relative protective activity of the effect of boosting with the bivalent vaccine or a natural infection with XBB.1.5.

### Boosting with the bivalent vaccine protects against lung inflammation caused by SARS-CoV-2 XBB.1.5 infection in K18-hACE2 mice

**Figure 4:**
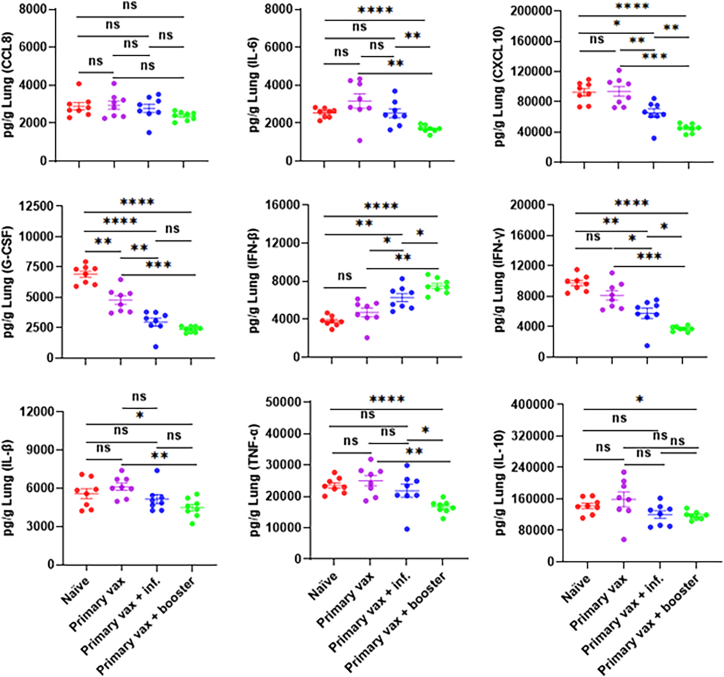
SARS-CoV-2 XBB.1.5-induced lung inflammation reduced by boosting with bivalent mRNA vaccine Cytokine and chemokine protein levels in lung homogenates at 4 dpi were quantified by ELISA. Data are represented as means (pg/g lung) ±SEM (*n* = 8 per group). Horizontal lines indicate mean values. ns, not significant; ∗*p* < 0.05, ∗∗*p* < 0.01, ∗∗∗*p* < 0.001, and ∗∗∗∗*p* < 0.0001.

Increased levels of cytokine and chemokine concentrations during the acute phase of COVID-19 are associated with an elevated risk of disease severity and mortality.^31^^,^^32^^,^^33^ As an additional indicator of vaccine-mediated protection, we next evaluated whether vaccination and boosting with the bivalent mRNA vaccine, or prior intranasal infection with XBB.1.5, could suppress cytokine and chemokine levels in the lungs at 4 dpi following challenge with XBB.1.5 in K18-hACE2 mice (Figure 4). Proinflammatory cytokines and chemokines including interleukin (IL)-6, chemokine (C-X-C motif) ligand 10 (CXCL10), granulocyte colony-stimulating factor (G-CSF), interferon-gamma (IFN-γ), interleukin-1β (IL-1β), tumor necrosis factor alpha (TNF-α), and interleukin-10 (IL-10), were significantly lower in animals that were vaccinated and boosted with the bivalent mRNA vaccine compared to unvaccinated control animals (Figure 4). While animals that received the bivalent mRNA vaccine and were intranasally infected with XBB1.5 exhibited significantly lower levels of CXCL10, G-CSF, and IFN-γ, some of the chemokines and cytokines measured including IL-6, IL-1β, TNF-α, and IL-10 were similar to those in mice that that received only the primary vaccination series with the bivalent mRNA or in naive control mice that were not vaccinated (Figure 4). IFN-β was significantly elevated in all vaccinated groups compared to unvaccinated controls, with the highest levels observed in the vaccinated and boosted (Figure 4). Notably, this occurred despite undetectable infectious virus and low viral RNA levels, suggesting that IFN-β induction may result from rapid innate sensing of residual viral antigens. In contrast, IFN-γ expression was significantly reduced in both the vaccinated and boosted and the vaccinated and infected groups relative to the unvaccinated controls, consistent with decreased need for Th1-mediated inflammation in the context of stronger viral control. In line with the virological data, protection against breakthrough infection and XBB.1.5-induced inflammation was substantially enhanced by boosting with the bivalent mRNA vaccine compared to exposure to XBB.1.5 infection following a primary vaccination series or just the primary vaccination series.

## Discussion

The rapid evolution and immune escape of SARS-CoV-2 has diminished the protective efficacy of the current vaccines against emerging variants, necessitating the use of regular boosters and urgent development of updated vaccines that are effective against circulating variants. Beginning in September 2022, bivalent mRNA vaccines targeting both the ancestral D614G spike protein and the Omicron BA.1 or BA.4-5 spike proteins were administered as booster doses. By September 2023, a monovalent Omicron XBB.1.5 vaccine was rolled out to enhance the production of neutralizing antibodies against the latest Omicron variants. In late fall 2024, the XBB.1.5 was replaced with the monovalent JN.1 vaccine to target most recent variants (https://covid19.who.int). The ongoing circulation of diverse SARS-CoV-2 variants, combined with different vaccine types and prior infections, has led to a wide range of immune responses within the population. This variability presents challenges in developing effective vaccination strategies and in accurately assessing the risks posed by emerging variants, as well as the potential immunogenicity and protective efficacy of updated vaccines across different vaccination regimens (https://www.ema.europa.eu/en/news/etf-concludes-bivalent-original-omicron-ba4-5-mrna-vaccines-may-be-used-primary-vaccination). Preclinical animal models of COVID-19 remain necessary to evaluate the immunogenicity and protective efficacy of updated vaccines against circulating variants, particularly in naive animals, as most of the global population has already been exposed through infection or vaccination.^34^^,^^35^^,^^36^^,^^37^ In this study, we assessed the immunogenicity and protective efficacy of the bivalent (Original/Omicron BA.4-5) mRNA vaccine against SARS-CoV-2 XBB.1.5 breakthrough infection in K18-hACE2 transgenic mice, using various vaccination schedules. Mice were primed with the bivalent vaccine to evaluate its full immunogenic potential in a naive setting, free from prior antigenic imprinting or confounding diverse and complex immune profiles seen in humans. The aim of this study was to assess the intrinsic potential of the bivalent vaccine to generate broad and protective responses against antigenically distant variants such as XBB.1.5.

We first evaluated the immunogenicity of the vaccine administered either as a two-dose primary vaccination series, or booster vaccination (three-dose series), or a primary vaccination followed by an intranasal XBB.1.5 infection. Booster vaccination (three-dose series) with the bivalent vaccine induced significantly stronger neutralizing antibody responses against both the XBB.1.5 and Wuhan D614G strains compared to the primary vaccination group. Since the bivalent vaccine targets the wild-type (WT, D614G) strain (Pfizer-BioNTech), boosting with the bivalent vaccine resulted in a stronger neutralizing antibody response against the Wuhan D614G strain than against the XBB.1.5 variant. These findings suggest that additional doses of the bivalent vaccine increase neutralizing antibody strength against both the historical Wuhan D614G and the XBB.1.5 viruses. This aligns with human serum data obtained after bivalent vaccines (as a fourth booster) targeting Omicron SARS-CoV-2 variants, with the exception of XBB.1 strains, which showed diminished neutralization capacity against XBB.1 strains.^1^^,^^2^^,^^3^ While our results show robust neutralizing responses in sera against XBB.1.5 when the bivalent vaccine is administered three times, these responses are still markedly lower than the responses against virus strains that are matched to the immunization antigen. This is likely because of the large number of mutations in the XBB.1.5 spike protein, which allow the variant to partially escape neutralizing antibodies generated against the Wuhan-1 and even BA.4/5 spike antigens.^9^^,^^10^^,^^11^ While most humans today are not naive to SARS-CoV-2, these findings in a naive animal model demonstrate that a homologous three-dose series of the bivalent mRNA vaccine can significantly boost antibody responses against antigenically distant variants, such as XBB.1.5. These results underscore the immunological benefit of homologous boosting with updated bivalent vaccines in a controlled model system, and may offer insight into how antigenic distance affects booster efficacy in the absence of prior imprinting. This is consistent with predictive models in humans predicting that boosting with variant-modified vaccines can improve protection, even when not perfectly matched to circulating variants.^38^ Although intranasal infection after vaccination improved neutralization compared to two doses, it was inferior to three-dose boosting. Our results highlight the now well-established understanding that, even in the event of a breakthrough infection following vaccination, a booster may still be necessary to enhance neutralizing activity.^39^ This observation is consistent with human studies demonstrating that triple immunization can induce high-quality antibodies with superior neutralization capacity against emerging variants, and that Omicron breakthrough infections are less immunogenic, providing reduced protection because higher antibody titers are observed in severe versus mild breakthrough infection.^40^^,^^41^^,^^42^ Due to vaccine-induced protection, viral replication might have been restricted resulting in limited antigen load when mice were boosted with an intranasal infection of XBB.1.5. This likely resulted in weaker stimulation of systemic immunity compared to the controlled and robust antigen exposure delivered by a third mRNA vaccine dose.

Regardless of the vaccination schedule, immunization with the bivalent mRNA vaccine effectively prevented weight loss and conferred complete protection against a sublethal XBB.1.5 challenge in K18-hACE2 mice. No infectious virus was recovered from the lungs of vaccinated mice. Animals that received a booster vaccination of the bivalent vaccine (three-dose series) or a primary two-dose series and infected with XBB.1.5 exhibited significantly higher protection against lung infection (∼6 log copies/g viral RNA reduction relative to naive animals) compared to animals that only received a primary two-dose bivalent vaccine (∼4 log copies/g reduction relative to naive animals). Since non-viable virus was detected in the lungs of all vaccinated animals, compared to the naive animals, one possible interpretation is that abortive SARS-CoV-2 replication occurred. This suggests that breakthrough infections from vaccinated mice maybe less infectious. However, the significantly reduced lung viral RNA in the “primary two-dose series + XBB.1.5 infected” group supports the notion of rapid immune-mediated clearance rather than absence of viral exposure. However, without direct confirmation of productive infection immediately after (e.g., 1–4 days post infection) or measurements of mucosal immune responses (e.g., sIgA or lung-resident memory T cells), it remains unclear whether the intranasal exposure served as a true immunological booster or simply re-engaged systemic memory responses at a low level. The comparatively lower neutralizing titers in this group, relative to the three-dose vaccinated mice, may reflect differences in Spike protein antigen load and presentation. Future studies including bronchoalveolar lavage fluid analysis and mucosal immunophenotyping would help clarify the extent and nature of immune activation in breakthrough exposures. Our data are consistent with human studies showing that booster vaccinations or infection after vaccination seems to reduce viral loads and infectiousness in subsequent breakthrough infections with SARS-CoV-2 variants.^15^^,^^36^^,^^43^^,^^44^

Our data also demonstrated that vaccination with the bivalent mRNA vaccine elicited robust T cell immune responses and S protein-specific CD8^+^ T cells. This is consistent with human studies indicating that memory T cells play a key role in controlling of SARS-CoV-2 infection and replication, and that bivalent mRNA vaccine-elicit SARS-CoV-2 specific T cells are capable of recognizing the XBB sublineage.^45^^,^^46^

A key goal of effective vaccination is not only to prevent viral replication but also to limit pathological inflammation. Elevated cytokines IL-6, IFN-γ, and TNF-α have been associated with severe COVID-19 outcomes.^31^^,^^32^^,^^33^ In this study, boosting with the bivalent mRNA vaccine led to significantly reduced lung cytokine and chemokine levels after XBB.1.5 challenge, compared to unvaccinated controls and two-dose vaccinated mice. This reduction reflects better control of both viral replication and downstream inflammation. The vaccinated and XBB.1.5-infected group showed partial cytokine suppression, but key inflammatory markers (e.g., IL-6 and IL-1β) remained elevated suggesting less effective immune modulation compared to homologous boosting. Interestingly, IFN-β was elevated in all vaccinated groups despite minimal viral replication, possibly indicating innate sensing or immune recall.^47^ In contrast, IFN-γ was reduced in vaccinated and boosted, and vaccinated and XBB.1.5 exposed groups, consistent with lower need for inflammatory Th1 responses in well-protected hosts.^12^^,^^13^ These data show that bivalent vaccine boosting not only enhances immunity but also dampens lung inflammation after XBB.1.5 challenge.

In summary, our study demonstrates that increasing doses of the bivalent (ancestral and BA.4/5) mRNA vaccine can induce potent and protective cellular and humoral responses against SARS-CoV-2 XBB.1.5, an antigenically distinct Omicron variant. These findings also support the interpretation that while initial vaccine-induced immunity likely limited viral replication, intranasal exposure to XBB.1.5 nonetheless provided some antigenic stimulation to boost the immune response. Our experimental rationale reflects real-world challenges: in humans, breakthrough infections post-vaccination especially with Omicron variants are less immunogenic and provide reduced protection against reinfection.^42^ However, subclinical or rapidly cleared infections may still serve as immunological boosters.^48^^,^^49^ While our study was conducted in a naive mouse model, the observed immunological enhancement following a third dose of the bivalent vaccine suggests that homologous boosting may still be relevant to populations with limited pre-Omicron exposure, including children born during the Omicron era and immunologically naive individuals in regions with delayed vaccine rollout. Future studies incorporating models of ancestral imprinting will be important to evaluate how immune history shapes the efficacy of variant-updated vaccines and to better inform vaccine strategies in a globally exposed population. Evaluating the efficacy of currently available booster vaccines against emerging SARS-CoV-2 variants remains an important strategy for addressing future COVID-19 challenges.

### Limitations of the study

This study was conducted in K18-hACE2 transgenic mice, a model that supports robust SARS-CoV-2 replication and disease but may not fully recapitulate human immune responses or the effects of prior antigenic imprinting. While this naive model allows controlled evaluation of vaccine- and infection-induced immunity, it does not reflect the complex immunological histories of most human populations. Productive infection following intranasal XBB.1.5 exposure was not directly confirmed by early post-infection time points or mucosal immune readouts, such as secretory IgA or lung-resident memory T cells. Consequently, it remains unclear whether viral exposure acted as a true immunological booster or merely re-engaged systemic immune memory, which is a likely scenario in the majority of the human population. Additionally, we did not compare the bivalent vaccine with monovalent XBB.1.5 boosters, which limits interpretation regarding current variant-matched vaccines. Future studies incorporating mucosal immune profiling, imprinting models, and direct comparisons with updated monovalent boosters will be important to refine vaccine strategies against emerging SARS-CoV-2 variants.

## Resource availability

### Lead contact

Requests for further information and resources should be directed to and will be fulfilled by the lead contact, Sebenzile K. Myeni (s.k.myeni@lumc.nl).

### Materials availability

This study did not generate new unique reagents.

### Data and code availability

• The published article includes all relevant dataset generated or analyzed during this study. Accession numbers are listed in the key resources table.
• This paper does not report original code.
• All data reported in this paper will be shared by the lead contact upon request.

## Consortia

The members of BREAK COVID group are Ramon Arens, Sandra de Bruin-Versteeg, Mo Arkani, Stefan A. Boers, Anja Garritsen, Jelle J. Goeman, Simon P. Jochems, Szymon M. Kielbasa, Sander de Kivit, Rajagopal A. Murugan, Sebenzile K. Myeni, Nicole A. Rogowski, Ed D.L. Schmidt, Eric J. Snijder, Igor Sidorov, Frank J.T. Staal, Jutte J.C. de Vries, Rory de Vries.

## Acknowledgments

We thank the LUMC Experimental Animal Facility, especially Marleen Blom, Marloe Pijnacker-Verspuij, Ewoud Speksnijder, and Jos van der Kaa. We also thank Yvonne de Vaal for her T cell analysis expertise. This publication is part of the project BREAK COVID with project number 10430072110009 of the COVID-19 research program that is (partly) financed by the 10.13039/501100001826Netherlands Organisation for Health Research and Development (ZorgOnderzoek Nederland Medical Sciences -ZonMw).

## Author contributions

Conceptualization, M.E.L., S.H.T.J., J.B.t.H., and S.K.M.; methodology, M.E.L., S.H.T.J., J.B.H., F.R., E.C., and J.C.Z.-D.; investigation, M.E.L., S.H.T.J., J.B.H., F.R., E.C., and J.C.Z.-D.; writing – original draft, M.E.L., S.H.T.J., and S.K.M.; writing – review and editing, M.E.L., S.H.T.J., R.M., J.J.C.d.V., and S.K.M.; funding acquisition, S.K.M. and J.J.C.d.V.; supervision, S.K.M. All authors have read and agreed to the published version of the manuscript.

## Declaration of interests

The authors declare no competing interests.

## STAR★Methods

### Key resources table

**Table:** 

REAGENT or RESOURCE	SOURCE	IDENTIFIER
**Antibodies**		

Goat anti-mouse IgG-HRP (Jackson Immuno Research)	Jackson Immuno Research	RRID: AB_2313585
CD3-BV510	BD Biosciences	Clone 145-2C11; RRID: AB_394591
CD44-BV786	BioLegend	clone IM7, cat 103059; RRID: AB_2571953
CD8a-BV605	BioLegend	Clone 53-6.7, cat 100743; RRID: AB_2561352
CX3CR1-BV785	BioLegend	Clone SA011F11, cat 149029; RRID: AB_2565938
KLRG1-BV785	BioLegend	Clone 2F1, cat 138429; RRID: AB_2629749
CD62L-BV421	BioLegend	Clone MEL-14, cat 104435; RRID: AB_10900082
CD44-BV786	BioLegend	clone IM7, cat 103059; RRID: AB_2571953

**Bacterial and virus strains**		

SARS-CoV-2/Leiden-008	Leiden University Medical Center	GenBank: MT705206.1

**Biological samples**		

Bivalent (original and Omicron BA.4/BA.5) mRNA COVID-19 vaccine	Pfizer-BioNTech	Comirnaty Original/Omicron BA.4-5

**Chemicals, peptides, and recombinant proteins**		

PepTivator SARS-CoV-2 Prot_S	Miltenyi Biotec	130-127-953
PepTivator SARS-CoV-2 Prot_N	Miltenyi Biotec	130-126-699
PepTivator SARS-CoV-2 Prot_S B.1.1.529/BA.2 mutation pool	Miltenyi Biotec	130-130-806
SARS-CoV-2 Spike protein of Wuhan	R&D systems	10561-CV-100
SARS-CoV-2 Spike protein Omicron BA.2 variant	R&D systems	11109-CV-100
Spike 539-546, VNFNFNGL, MHC-I tetramer to identify S-specific CD8^+^ T cells	Generated inhouse (Leiden University Medical Center)	VNFNFNGL
Brefeldin A (Golgiplug)	BD Biosciences	555029

**Critical commercial assays**		

mouse IFN-γ ELISpot-plus kit	Mabtech	3321-4APW-10
Mouse IFN-beta DuoSet ELISA	R&D Systems	DY8234-05
Mouse TNF-α DuoSet ELISA	R&D Systems	DY410-05
Mouse IL-6 DuoSet ELISA	R&D Systems	DY406-05
Mouse IFN-γ DuoSet ELISA	R&D Systems	D485-05
Mouse CXCL10 DuoSet ELISA	R&D Systems	DY466-05
Mouse IL-1β DuoSet ELISA	R&D Systems	DY401-05
Mouse IL-10 DuoSet ELISA	R&D Systems	DY417-05
Mouse CCL8 DuoSet ELISA	R&D Systems	DY790
Mouse G-CSF DuoSet ELISA	R&D Systems	DY414

**Experimental models: Cell lines**		

VeroE6	Master stock MM-3 from the Dept. of Medical Microbiology, Leiden University Medical Center) collection, characterized by full-genome sequencing	
Calu-3	ATCC	#HTB-55

**Experimental models: Organisms/strains**		

K18-hACE2 transgenic mice	Jackson Laboratory	B6.Cg-Tg(K18-ACE2)2Prlmn/J

**Oligonucleotides**		

forward- GTGARATGGTCATG TGTGGCGG - RdRp_Sarbeco	IDT Integrated DNA Technologies	forward- GTGARATGGTCATGT GTGGCGG - RdRp_Sarbeco
reverse-CARATGTTAAASACAC TATTAGCATA–RdRp_Sarbeco_R	IDT Integrated DNA Technologies	reverse-CARATGTTAAASACACTATT AGCATA–RdRp_Sarbeco_R
FAM-CCAGGTGGAACMTCATCMG GWGATGC-BHQ1-RdRp_Sarbeco_Probe)	IDT Integrated DNA Technologies	FAM-CCAGGTGGAACMTCATCMGG WGATGC-BHQ1-RdRp_Sarbeco_Probe)
forward-ACAGGTACGTTAATAGTTA ATAGCGT–E_Sarbeco_F	IDT Integrated DNA Technologies	forward-ACAGGTACGTTAATA GTTAATAGCGT–E_Sarbeco_F
reverse- ATATTGCAGCAGTACG CACACA–E_Sarbeco_R	IDT Integrated DNA Technologies	reverse- ATATTGCAGCAGTACGC ACACA–E_Sarbeco_R
TexRed-ACACTAGCCATCCTTACTGC GCTTCG-BHQ2	IDT Integrated DNA Technologies	TexRed-ACACTAGCCATCCTTA CTGCGCTTCG-BHQ2

**Software and algorithms**		

GraphPad Software version 9	Boston, Massachusetts USA	www.graphpad.com
Flowjo software (TreeStart)	FlowJo, LLC	
OMIQ data analysis software	OMIQ	https://www.omiq.ai/
Aurora Cytek spectral analyzer with SpectroFlo software version 3	Cytek Biosciences	
BD FACS DIVA software (version 9)	BD Biosciences	

### Experimental model and study participant details

#### Mouse model

K18-hACE2 transgenic mice, which express the human ACE2 receptor under the control of the cytokeratin 18 (K18) promoter^50^ were purchased from the Jackson Laboratory (B6.Cg-Tg(K18-ACE2)2Prlmn/J) and bred at the LUMC Central Animal Facility (PDC). All experiments involving mice were reviewed and approved by the Animal Experiments Committee of the LUMC and performed according to the recommendations and guidelines set by LUMC, the Dutch Experiments on Animals Act and in strict accordance with EU regulations (2010/63/UE).

#### Cell lines

Calu-3 cells (ATCC, #HTB-55) were cultured at 37°C in minimum essential medium with Earle’s salts (EMEM, Gibco) supplemented with 10% FCS, 2 mM L-glutamine, 0.1 mM non-essential amino acids (Lonza), 1 mM sodium pyruvate (Thermo Fisher Scientific), 50 IU/ml penicillin, and 50 μg/ml streptomycin. VeroE6 cells were grown in Dulbecco’s modified Eagle’s medium (DMEM; Lonza) supplemented with 8% fetal calf serum (FCS; Bodinco BV), 50 UI/mL penicillin (Lonza) and 50 μg/ml streptomycin (Lonza) at 37°C and 5% CO_2_. All cells were routinely tested for negative mycoplasma using a PCR-based assay.

#### Viruses

Clinical isolates SARS-CoV-2/Leiden-008 and SARS-CoV-2/XBB.1.5 were isolated from nasopharyngeal samples and NGS sequenced. The sequence of the SARS-CoV-2/Leiden-008 is available under GenBank accession number MT705206.1. Compared to the original Wuhan isolate, the SARS-CoV-2/Leiden-008 contains the D614G mutation in the spike protein and three additional non-silent mutations, namely C12846U in nsp9 (A54V), C14408U in nsp12 (P323L), and C18928U in nsp14 (P267S). All viruses were passaged in Calu-3 cells, titrated by plaque assay on VeroE6 cells and subjected to next-generation sequencing. All experiments with live SARS-CoV-2 were performed in an approved biosafety level 3 (BSL-3) laboratory at Leiden University Medical Center (LUMC).

### Method details

#### Infection of K18-hACE2 mice with SARS-CoV-2

K18-hACE2 transgenic mice, which express the human ACE2 receptor under the control of the cytokeratin 18 (K18) promoter^50^ were purchased from the Jackson Laboratory (B6.Cg-Tg(K18-ACE2)2Prlmn/J) and bred at the LUMC Central Animal Facility (PDC). All experiments with infected animals were performed in a class 3 biological safety cabinet in the Animal BSL3 unit of the LUMC Central Animal Facility (DM3 unit). Mice were housed in individually ventilated isolator cages (IsoCage Biocontainment System, Tecniplast) under specified pathogen free conditions with *ad libitum* access to food and water and cage enrichment at 20°C -22°C, a humidity of 45-65% RV and a light cycle of 6:30h-7:00h sunrise, 07:00h-18:00h daytime and 18:00h-18:30h sunset. Male and female mice aged 8-12 weeks at the start of the experiment were pulled from several independent litters and randomized over experimental groups, and after transfer to the ABSL-3 facility, acclimated for a period of 7 days prior to the start of the experiments. Virus inoculations were performed under isoflurane anesthesia and infected mice were monitored daily for weight loss, disease symptoms and survival. Mice that reached 20% weight loss were deemed to have reached their humane endpoints and euthanized. Mice that were considered moribund were euthanized at the discretion of the researcher and designated veterinarian. The endpoint of survival experiments was set at 14 days post inoculation (p.i.), except for mice that died or reached a humane endpoint before that time. At various designated time points, mice were euthanized with an overdose of sodium pentobarbital (Euthasol 200 mg/kg, injected intraperitoneally under isoflurane anesthesia), and tissue samples were collected for determining virus titration/immune responses.

#### Immunization and challenge of K18-hACE2 mice

For immunization, male and female mice, aged 8-12-weeks, were randomized into four groups (group 1 – Naive, group 2 – Primary vax, group 3 – Booster vax and group 4 – Primary vax + infection). The bivalent (original and Omicron BA.4/BA.5) mRNA COVID-19 vaccine (Pfizer-BioNTech) vaccine was diluted in 0.9% NaCl, and 1 μg of the vaccine candidate was administered intramuscularly, in the thigh of one of the hind limbs at a volume of 20 μl under isoflurane anesthesia using a two-week interval prime-boost regimen. Third immunization (booster) with the bivalent mRNA vaccine (1 μg , intramuscularly) or intranasal infection with XBB.1.5 (10^4^ PFU) occurred at day 63 after study start. This viral dose was selected based on unpublished titration data in the K18-hACE2 mouse model showing it produces robust viral replication and disease in naïve animals, and was also used for challenge studies. Blood sampling for serum isolation for binding and neutralizing antibody determination was collected on specified days via tail-cut vein under isoflurane anesthesia. For serum generation, blood was centrifuged for 5 min at 16,000*g* and the serum was immediately used for downstream assays or stored at −20°C until time of use. Spleens were collected at day 91 and 116 since the start of the study. Five weeks after the booster immunization (day 98 since the start of the study), naïve and immunized mice were challenged intranasally (i.n.) with SARS-CoV-2/XBB.1.5 (10^4^ PFU in 50 μL DMEM). Morbidity/mortality status and weights were assessed and recorded daily for 14 days. At days 0, 2, 4, 6 and 14 oral swabs were collected and at day 4 post challenge, tissues were harvested for virological analyses (virus titration by plaque assay, RT-PCR and immune responses).

#### Lung virus titers

To determine virus titers, lungs were weighed and placed in a gentleMACS M Tube (Miltenyi Biotec) containing 2 ml of PBS with 100 units/mL penicillin, 100 units/mL streptomycin (Lonza), 50 μg/ml gentamycin (Sigma-Aldrich), and 0.25 μg/ml Fungizone (Gibco). Lung tissues were homogenized with the gentleMACS dissociator by running program Lung_02 (Miltenyi Biotec, Inc.). Supernatants from homogenized samples were pre-clarified at 300 xg for 1 min and then further centrifuged at 10 000RPM for 5 min. Thereafter, infectious virions in the lung were determined by plaque assay on VeroE6 cells and expressed as PFU/g lung. The quantification of SARS-CoV-2 viral RNA was performed using lung homogenates lysed with TriPure isolation reagent (Roche Applied Science) in gentleMACS M tubes (Miltenyi Biotec) according to the manufacturer’s instructions. RNA was extracted by the addition of chloroform and liquid phases were separated by centrifugation. RNA was then precipitated from the aqueous phase using isopropanol. SARS-CoV-2 viral copy numbers were determined by RT-qPCR using the TaqMan Fast Virus 1-step master mix (thermos Fisher Scientific) on a CFX384 Touch Real-Time PCR Detection System (BioRad). The sub-genomic mRNA PCR primers and probes against the E gene and the genomic RdRp gene were modified based on previously described primer and probe sets (Corman *et al*, 2020). The primers and probes were as follows; the sub-genomic mRNA PCR primers (forward- GTGARATGGTCATGTGTGGCGG - RdRp_Sarbeco_F) and reverse-CARATGTTAAASACACTATTAGCATA–RdRp_Sarbeco_R) and probe (FAM-CCAGGTGGAACMTCATCMGGWGATGC-BHQ1-RdRp_Sarbeco_Probe) and the genomic RNA PCR primers (forward-ACAGGTACGTTAATAGTTAATAGCGT–E_Sarbeco_F) and (reverse- ATATTGCAGCAGTACGCACACA–E_Sarbeco_R) and probe (TexRed-ACACTAGCCATCCTTACTGCGCTTCG-BHQ2) were used. A standard curve was obtained using an *in vitro* transcript derived from a synthetic plasmid that contained all PCR targets. Each RNA sample was analyzed in triplicate.

#### Live SARS-CoV-2 neutralization assay

VeroE6 cells were seeded at 12,000 cells/well in 96-well tissue culture plates 1 day prior to infection. Heat-inactivated (30 min at 56°C) serum samples were analyzed in duplicate. The panel of sera were two-fold serially diluted in duplicate, with an initial dilution of 1:10 and a final dilution of 1:8240 in 60 μL EMEM medium supplemented with penicillin, streptomycin, 2 mM L-glutamine and 2% FCS. Diluted sera were mixed with equal volumes of 120 TCID_50_/60 μL SARS-CoV-2 and incubated for 1 h at 37°C. The virus-serum mixtures were then added onto VeroE6 cell monolayers and incubated at 37 C and 5% CO_2_. Cells either unexposed to the virus or mixed with 120 TCID_50_/60 μL SARS-CoV-2 were used as negative (uninfected) and positive (infected) controls, respectively. At 3 days post-infection (for SRS-CoV-2/Leiden-008 D614G) and 4 days post-infection (for SARS-CoV-2/XBB.1.5), cells were fixed and inactivated with 40μL 37% formaldehyde/PBS solution/well overnight at 4 C. The fixative was removed from cells and the clusters were stained with 50 μL/well crystal violet solution, incubated for 10 min, and rinsed with water. Dried plates were evaluated for viral cytopathic effect. The virus neutralization titer was expressed as the reciprocal value of the highest dilution of the serum, which still inhibited virus replication. A SARS-CoV-2 back-titration was included with each assay run to confirm that the dose of the used inoculum was within the acceptable range of 30 to 300 TCID_50_.

#### Enzyme-linked immunosorbent assay (ELISA)

High binding half-area 96-well ELISA plates (Corning Inc.) were coated with SARS-CoV-2 Spike protein of Wuhan (10561-CV-100, R&D systems) and Omicron BA.2 variant (11109-CV-100, R&D systems) at 25 ng/well in 0.M sodium carbonate buffer (pH 9.6) overnight at 4°C. Coated plates were washed three times with washing buffer (phosphate buffered saline with 0.05% Tween 20) and incubated with 5% skimmed milk for 1 h at RT for blocking. After washing three times, plates were incubated 20 μL of 8-step 1:2 serially diluted sera with a starting dilution of 1:2000 for 2h at RT. Upon washing, S protein specific antibodies were detected using goat anti-mouse IgG-HRP (Jackson Immuno Research) at 1:1000 in 0.5% skimmed milk in PBS with 0.05% Tween 20 and 1-step TMB substrate solutions (ThermoFischer Scientific 34021). Measured absorbance values were normalized using IgG1 standard curve (BD Pharming) with known concentration on each plate.

Clarified lung homogenates also used for virus titration as described above were used to evaluate the levels of interferons and cytokines. The tested samples were diluted at 1:2 in diluent buffer provided for each ELISA kit and added to each well, and 3 multiple wells were set for each sample. ELISA kits specific for mouse IFN-β (DY8234-05, R&D Systems), TNF-α (DY410-05, R&D Systems), IL-6 (DY406-05, R&D Systems), IFN-γ (D485-05, R&D Systems), CXCL10 (DY466-05), IL-10 (DY417-05), CCL8 (DY417-05), G-CSF (DY414) and IL-1β (D401-05, R&D Systems) were used following the manufacturer’s specifications.

#### ELISpot and flow cytometry

IFN-γ ELISpot was performed on mouse splenocytes isolated from vaccinated mice at 4 weeks post immunization using a mouse IFN-γ ELISpot-plus kit (Mabtech). Splenocytes were obtained by mechanically dissociating spleens through a sterile cell strainer and restimulated for 18–20 h at 37°C with a pool of PepTivator SARS-CoV-2 Prot_S containing the sequence domains aa 304-338, 421-475, 492-519, 683-707, 741-770, 785-802, and 885 – 1273 (Miltenyi Biotec) or aa 539-546 peptide pool at a final concentration of 1 μg/peptide/mL. Each sample of splenocytes was plated in triplicate wells and spots were developed using mouse IFN-γ ELISpot-plus kit (Mabtech), following the manufacturer’s instructions. The total numbers of spots in each well were counted using an ELISpot reader and converted into the number of spots per 1 million splenocytes for each well. The medium/unstimulated splenocytes were used as negative controls and a CD3/CD28 mix (dilution 1:150) was used as a positive control. IFN-γ-secreting splenocytes were reported as the average of spot forming cells (SFCs) per million splenocytes for each sample.

To assess intracellular cytokine production, single-cell suspensions (obtained by mincing the spleen tissue through cell strainers and depleting erythrocytes using ammonium chloride lysis buffer) were stimulated with peptides for 5 hours in the presence of brefeldin A (Golgiplug; BD Pharmingen). Flow cytometry was conducted using a BD Fortessa flow cytometer (BD Biosciences) with BD FACS DIVA software (version 9) or an Aurora Cytek spectral analyzer with SpectroFlo acquisition software (version 3). Data analysis was performed using FlowJo software (TreeStar) and OMIQ data analysis software.

### Quantification and statistical analysis

Data of biological replicates were reported for all experiments. All statistical analyses were performed using GraphPad Prism. Data are presented as mean ± standard error of the mean (SEM) or geometric mean ± geometric standard deviation (SD), as specified in figure legends. For comparisons between two groups, unpaired two-tailed Student’s *t*-tests were used. For comparisons among multiple groups, one-way ANOVA with Tukey’s multiple comparisons test or two-way ANOVA was performed as appropriate. *P* values of ^∗∗∗∗^*p* < 0.0001, ^∗∗∗^*p* < 0.001, ^∗∗^*p* < 0.01 and ^∗^*p* < 0.05 were considered significant and ns, not significant.
