# Supplementary material for: Bivalent mRNA vaccine booster enhances immunity against XBB.1.5 more effectively than breakthrough infection in K18-hACE2 mice
Source: iScience. 2025 Sep 1;28(10):113479. doi: 10.1016/j.isci.2025.113479 (PMC12481084; doi:10.1016/j.isci.2025.113479)
Supplement: Document S1. Tables S1 and S2 [file mmc1.pdf]

iScience, Volume *28*

Supplemental information

Bivalent mRNA vaccine booster enhances immunity against XBB.1.5 more effectively than breakthrough infection in K18-hACE2 mice

Marissa E. Linger, Sytze H.T. Jorritsma, Jonna Bloeme-ter Horst, Jessika C. Zevenhoven-Dobbe, Finn Rijlaarsdam, Emil Colstrup, Macha Beijnes, Jutte J.C. de Vries, Ramon Arens, Rajagopal Murugan, Sebenzile K. Myeni, and in collaboration with BREAK COVID group

Supplemental Information

Supplemental Table S1. Correlation between antigen-specific T-cell responses and lung viral RNA levels in vaccinated K18-hACE2 mice.

Correlation	T cell responses (ELISPOT) vs Lung viral RNA (Primary vax) – against Omicron BA.2	T cell responses (ELISPOT) vs Lung viral RNA (Primary vax + inf.) – against Omicron BA.2	T cell responses (ELISPOT) vs Lung viral RNA (Primary vax + booster) – Omicron BA.2	T cell responses (ELISPOT) vs Lung viral RNA (Primary vax + inf.) – against Wuhan D614	T cell responses (ELISPOT) vs Lung viral RNA (Primary vax + inf.) – against Wuhan D614	T cell responses (ELISPOT) vs Lung viral RNA (Primary vax + booster) – against Wuhan D614
Spearman r	-1.000	-1.000	-0.9940	-0.9940	-1.000	-0.9940
P value	<0.0001	<0.0001	<0.0001	<0.0001	<0.0001	<0.0001
Significant	****	****	****	****	****	****
n	8	8	8	8	8	8

The correlation between T cell responses (measured by ELISPOT assay Fig. 2) and viral RNA levels in the lungs (measured at 4 dpi, Fig. 3e) across different vaccination groups. A strong inverse correlation (r ~ –1.0) was observed in all groups, indicating that higher T cell responses were associated with lower viral RNA levels. Spearman correlations were statistically significant (P < 0.0001), supporting the role of T cells in vaccine-induced protection. Each group included *n* = 8 mice.

Supplemental Table S2. Correlation between serum neutralizing antibody titers and lung viral RNA Levels in vaccinated K18-hACE2 Mice.

Correlation	Neutralizing antibodies vs Lung viral RNA (Primary vax) – against XBB.1.5	Neutralizing antibodies vs Lung viral RNA (Primary vax + inf.) – against XBB.1.5	Neutralizing antibodies vs Lung viral RNA (Primary vax + booster) – against XBB.1.5	Neutralizing antibodies vs Lung viral RNA (Primary vax) – against Wuhan D614	Neutralizing antibodies vs Lung viral RNA (Primary vax + inf.) – against Wuhan D614	Neutralizing antibodies vs Lung viral RNA (Primary vax + booster) – against Wuhan D614
Spearman r	-0.9512	-0.9940	-0.9695	-0.9880	-1.000	-0.9880
P value	<0.0018	<0.0001	<0.0006	0.0002	<0.0001	0.0002
Significant	**	****	***	***	****	***
n	8	8	8	8	8	8

The correlation between neutralizing antibody titers (Fig. 1d-e) and viral RNA levels in the lungs (measured at 4 dpi, Fig. 3e) across different vaccination groups. Strong inverse correlations (Spearman r = –0.95 to –1.00) were observed in all groups, indicating that higher neutralizing antibody titers were associated with lower viral RNA levels in the lungs. Spearman correlations were statistically significant ($P < 0.01$), supporting the contribution of neutralizing antibodies to protection in this model. Each group included n = 8 mice.
